# Hand (and orthopaedic) surgery in primitive war conditions

**DOI:** 10.1186/1753-6561-9-S3-A5

**Published:** 2015-05-19

**Authors:** Abdul Nasser Kaadan

**Affiliations:** 1 Department of Orthopaedics, Aleppo University, Syria

## 

Old Chinese and Egyptian physicians used some chemical methods for the treatment of wounds and injuries. Although the beneficial effect of passing surgical instruments through flame was well known to ancient civilizations, the heat as a preservative method in medical industry, was first introduced in 1809 in France. Joseph Lister (1827–1912) believed that it was microbes carried in the air that caused diseases to be spread in wards. Sterile gowns and caps were used by Gustav Neuber. Surgical masks were applied in 1897 by Mikulicz, while rubber gloves were advised by William Halstead in 1890.

For the last two and half years, Syria has suffered from the worst kind of war. So far more than 125 thousand civilians are dead and more than four hundred thousand injured. A lot of severe different casualties resulted, which are severely above the capacity of the local hospitals, especially since some hospitals were occupied by some fighting sides and used for military purposes. Some houses changed into field hospitals, where there is no degree of sterilization at all.

As I am an orthopedic surgeon, I found myself going back to practice a primitive form of sterilization. We applied smoking and boiling the metal surgical instruments. Other non-metal instruments were used without sterilization. Some surgical procedures, such as amputations, were performed with bare hands, as there are no surgical gloves. Bone saws were sterilized by flaming. The percentage of infection is very high due to a lack in antibiotics as well.

The aim of this paper is to shed light on the primitive methods of sterilization inside some hospitals in Syria, where the war is still flaming.

